# Assessment of myocardial perfusion using contrast echocardiography – Case report

**Published:** 2015

**Authors:** L Rotaru, T Nanea

**Affiliations:** * Ministry of Internal Affairs “Prof. Dr. D. Gerota” Emergency Hospital; “Carol Davila” University of Medicine and Pharmacy, Bucharest, Romania

**Keywords:** myocardial perfusion, microbubble contrast agents, contrast echocardiography

## Abstract

Contrast echocardiography is a technique that improves endocardial demarcation and provides real-time data on blood circulation (blood flow, velocity). Left ventricle imaging study using contrast agents that cross the pulmonary circulation allows an improved visualization of endocardial tissue. This creates a more accurate ultrasound evaluation of left ventricular dimensions and its kinetics. Contrast echocardiography can improve Doppler mode evaluation and can provide information on myocardial perfusion precisely through this mechanism.

Microbubble contrast agents are second-generation ultrasound contrast agents and are especially useful in endocardial demarcation. Second generation ultrasound contrast agents available now, include “Definity”, “Optison” - available in almost all countries with an average medical system except for Europe and “SonoVue” - available in most European countries. Contrast agents are represented by microbubbles between 1-10μm in diameter, containing a gas surrounded by a phospholipid membrane (SonoVue) or protein (Optison).

Because the microbubble ultrasound characteristics used are different from the characteristics of the surrounding tissue or blood elements and cardiac structures, their diffusion produce very strong acoustic signals, which are directly proportional to blood volume. Quantitative assessment of myocardial microcirculation is now possible due to the advancing techniques in contrast echocardiography, provided that the left ventricular cavity has an increased echogenicity compared with the surrounding myocardium (which has a lower blood volume).

## Introduction

**Contrast Agents**

Echogenicity and properties of second-generation ultrasound contrast agents are determined by the size of the microbubbles, the type of encapsulated gas, and the type of the material that forms the microbubbles (lipid or protein) which is different for every contrast agent. The ultrasonic properties of these microbubbles are different from the ultrasonic properties of blood cells and myocardial tissue and represent a characteristic function of nonlinear oscillation, which means they will reflect ultrasound not only at the fundamental frequency characteristic for the ultrasound source, but with a considerable increase of the frequency. In general, the more elastic the microbubble wall, the more tissue oscillation will be generated, so the ultrasound image will be improved. In contrast, microbubbles with a thin membrane can break when exposed to an environment with high intensity ultrasound signal [**1**]. The main types of contrast agents, and their characteristics are presented in **Table 1** [**2**]. 

**Table 1:** Types of contrast agents used in contrast echocardiography (ESC Guidelines 2009 on indications of contrast echocardiography)

	SonoVue6	Optison7	Luminity8
Gas	Sulphur hexafluoride	Perfluoropropane	Perfluoropropane
Bubble size	2–8 µm	3.0–4.5 µm	1.1–2.5 µm
Surface coating	Surfactant/ powder	Human albumin	Naturally occurring lipids
Contraindications and precautions			
Patients experiencing side effects in clinical trials (%)	11	17	8
Most frequent side effects in clinical trials	Headache (2.1%), nausea (1.3%), chest pain (1.3%), taste perversion (0.9%), hyperglycaemia (0.6%), injection site reaction 0.6%), paresthesia (0.6%), vasodilation (0.6%), injection site pain (0.5%).	Headache (5.4%), nausea and/ or vomiting (4.3%), warm sensation or flushing (3.6%), dizziness (2.5%).	Headache (2.0%), flushing (1.0%), back pain (0.9%). rash/ urticaria, wheezing/,allergic/anaphylaxis
Manufacturer	Bracco Diagnostics	GE Healthcare	Lantheus Medical Imaging (formerly Bristol-Myers Squibb)

**Fig. 1:**
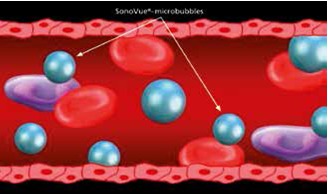
Schematic representation of the microbubbles in the vascular bed; (Braco: Cardiology SonoVue Monography)

At the moment, these are the European Society of Cardiology guidelines on contrast echocardiography:

a) In patients with **suboptimal ultrasound images at rest**

- In order to increase and improve endocardial visualization and evaluation of left ventricular structure and function when two or more segments are not well viewed in routine ultrasound examination [**3**]; 

- To take measurements with higher accuracy and reproducibility of left ventricular volumes and ejection fraction on 2D ultrasound exam;

- To confirm or exclude the echocardiographic diagnosis of structural abnormalities of the left ventricle, when the images are suboptimal, and an accurate diagnosis is needed in apical hypertrophic cardiomyopathy, ventricular noncompaction, apical thrombi, and ventricular pseudoaneurysms; 

b) **Together with the stress ultrasound test** when two or more limits of the left ventricular endocardium cannot be very well examined, in order to evaluate: ventricular wall motility and thickness in stress or at rest, to increase the frequency of diagnostic studies, to increase the exam reliability [**2**]. The real time assessment of myocardial perfusion is possible by using contrast echocardiography with second generation agents, because the size of these microbubbles is comparable to the size of a red blood cell (1-10 microns) and can penetrate the microcirculation up to the distal capillary [**3**] (**Fig. 1**).

Recent studies have found that the information and data accuracy obtained by using contrast echocardiography are similar with those offered by nuclear scintigraphy. Initial studies have shown that there is a good correlation between contrast echocardiography and regional imaging - SPECT (single photon emission computed tomography) in patients with coronary artery disease, but later it turned out that the myocardial contrast echocardiography obtained during exercise or other types of vasodilation inducing technique could bring additional data on the location of the affected area compared to SPECT [**4**].

**Fig. 2:**
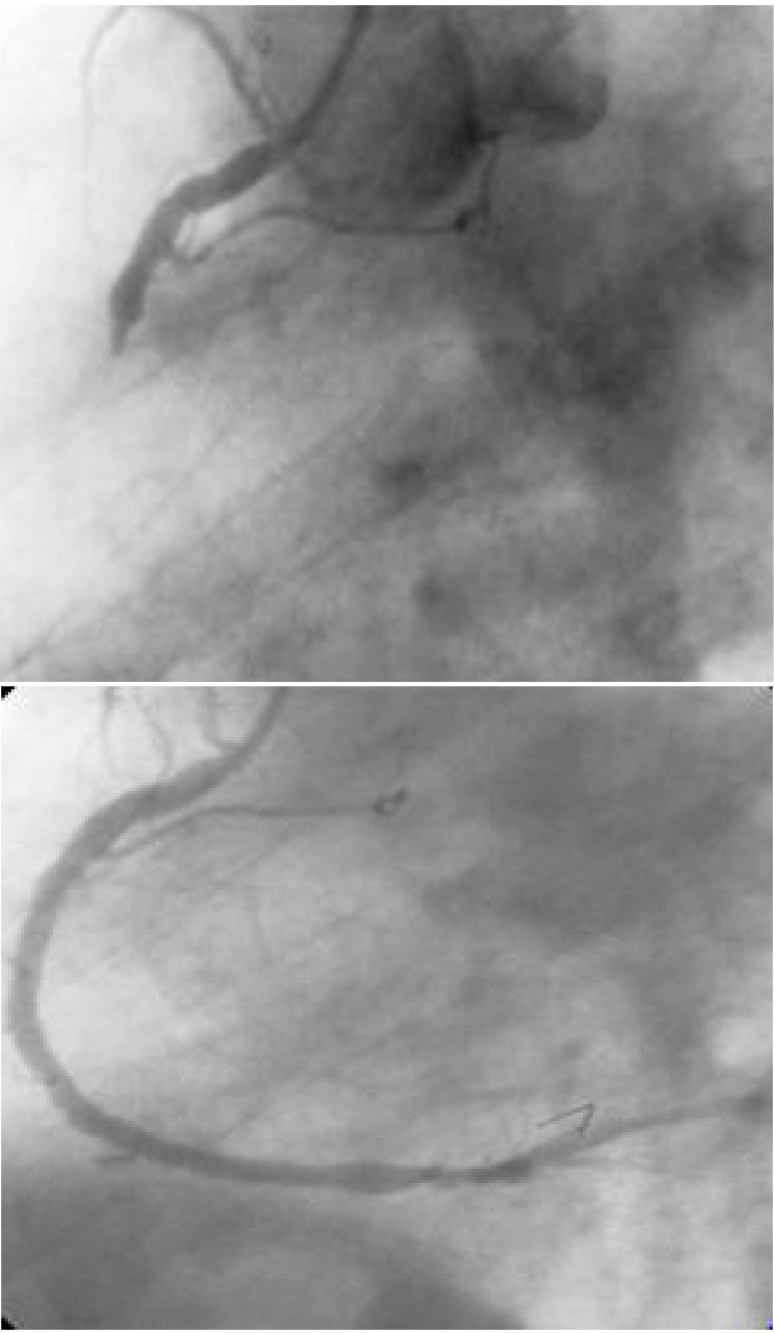
Myocardial blood flow quantification using contrast echocardiography; (ESC Guidelines 2009 on indications and usage of contrast echocardiography)

The fact that the degree of myocardial opacification is correlated with the blood volume present in the vascular territories on contrast echocardiography enables us to obtain information concerning the pathology of coronary arteries. The severity or the area affected by ischemia can be measured by using the product of myocardial flow velocity (the speed of capillary refill after their destruction with a special technique) and maximum intensity of the ultrasound wave (correlated with the blood volume in the studied tissue), thus obtaining extremely valuable additional data concerning the diagnosis and prognosis of coronary artery disease (**Fig. 2**).

Myocardial perfusion assessed by contrast echocardiography can bring additional information concerning the circulation and blood flow velocity compared to pulsed Doppler echocardiography or nuclear imaging by quantifying changes in blood flow (circulatory changes in response to stress). These changes can be identified by brightness and contrast differences. Circulatory changes that occur during stress or after the administration of vasoactive drugs, will expose ischemic myocardial tissue and will bring further details on blood circulation in that area. Compared to nuclear imaging, contrast echocardiography can be performed at bedside and enables immediate interpretation of the results [**5**].

In patients with acute myocardial infarction, contrast echocardiography performed before angioplasty can show the tissue at risk and, performed 24 hours after surgery (angioplasty), can identify the area where revascularization does not occur [**6**]. The use of contrast echocardiography in the evaluation of myocardial perfusion after primary angioplasty has a predictive value on outcome, concerning the efficiency of the revascularization in the left ventricle. The evaluation of myocardial perfusion in coronary microcirculation by contrast echocardiography is correlated with coronary flow reserve, evaluation that can also be performed by pulsed Doppler measurements [**7**].

Contrast echocardiography can provide prognostic data, as a survey (T. Sakuma [**8**]) of 50 patients who had myocardial infarction and received thrombolytic therapy, showed. The presence of lower intensity opacities (hypoechogenic) on the contrast echocardiography image, that have been performed on the second day after the administration of thrombolytic therapy, correlated with a higher recurrence of a heart attack or other severe heart pathologies (cardiac arrest, fatal or non-fatal heart attack and other cardiac pathologies) in the near future, on an average of 22 months from the first cardiovascular event (myocardial infarction). In another study, patients with a persistent defect in the infarct area due to unrecovered myocardial perfusion had regional or global systolic dysfunction [**9**,**10**].

## Case presentation

This is a case of contrast echocardiography assessment of a patient who is part of a group of 28 patients who presented myocardial infarction with ST-segment elevation (STEMI) and underwent stent angioplasty in coronary care centers in the first 6-12 hours from the onset of the acute symptoms. These were clinically assessed in a timeframe of 1-6 months post-infarction, and by ultrasound, including contrast echocardiography with contrast agent Sono-Vue (sulfur hexafluoride) by using Philips I33 system with the “Low IM” - low mechanical index program.

**Fig. 3:**
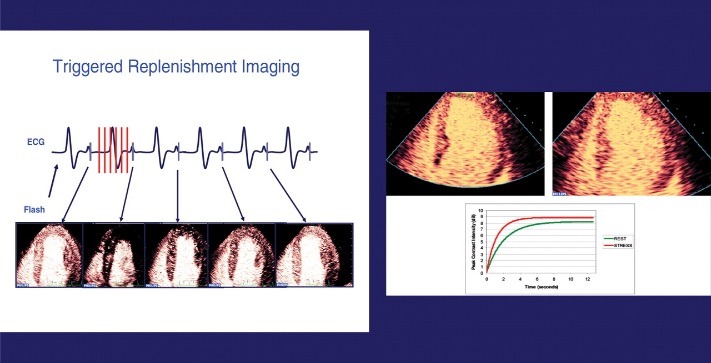
Coronary angiography that shows right coronary occlusion followed by stent angioplasty at this level (personal archive)

The patient RV aged 57 years old, obese, with modified fasting plasma glucose, presented an acute inferior ST elevated myocardial infarction. Approximately 6 hours after the symptomatic onset, the patient was evaluated in an intensive coronary care unit by coronary angiography. The investigation revealed right coronary artery occlusion, and stent angioplasty was successfully performed, with post-procedural TIMI 3 flow (**Fig. 3**). On admission, the T troponin was 8 times higher than the 99th percentile, mild leukocytosis was present, blood glucose level was 118mg/ dl, slightly elevated LDL cholesterol (198mg/ dl), serum triglycerides were 180 mg/ dl, and C-reactive protein - 0.5 mg/ dl. Left ventricular ejection fraction on echocardiography was 45-50% with a kinetic disorder in the posterior inferior ventricular wall - 5 of 17 segments with a 1.35 kinetics index.

**Fig. 4:**
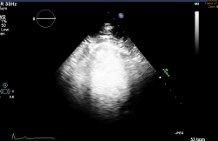
Contrast echocardiography - opacification of the left ventricle (performed with Philips IE33, LVO mode, personal archive)

A month after angioplasty, the patient was hemodynamically stable, with no angina, and was hospitalized for clinical and paraclinical assessment. Routine echocardiography and contrast echocardiography with Sono-Vue contrast agent (continuous intravenous injection - 1ml/ min for 3 minutes) were performed, showing reduced systolic parietal thickening of the myocardium on the posterior wall of the left ventricle, with a parietal kinetic score of 20 and a kinetic index of 1.17 (the normal value is 1) (**Fig. 4**); Ejection fraction measured by the Simpson method in apical 4 chamber and 2 chamber view was 53% (left ventricular end diastolic volume was 78 ml, left ventricular end systolic volume was of 36 ml). Left ventricular diastolic dysfunction with impaired relaxation pattern without mitral regurgitation without pulmonary hypertension was also present.

Myocardial perfusion study was performed by using the “low MI” low mechanical index (MI <0.2), this preset is used as microbubbles in the sound field strongly represented by IM> 0.5 being destroyed.

**Fig. 5:**
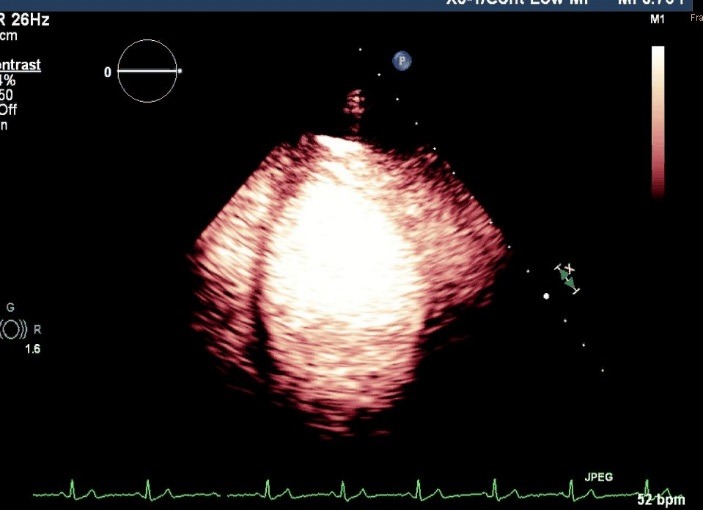
Contrast echocardiography, 4-chamber view - absent perfusion in the lower basal septum and heterogeneous uptake of the mid-third and apical segments (personal archive)

The study of myocardial perfusion showed a perfusion defect through the absence of the acoustic signal in the lower one third of the left ventricular wall, and a scratchy aspect in the middle third of the left ventricular wall after the destruction of the microbubbles with a strong signal (flash), and study of myocardial capillaries refill during continuous intravenous injection with SonoVue (1 ml/ min) - **Fig. 5**. The semiquantitative evaluation of the myocardial perfusion was performed by using the 17-segment model used in kinetic score and by evaluating the rate of contrast agent uptake in myocardial segments. The absence of the myocardial uptake was quantified as 0, heterogeneous or reduced uptake by 1 and the normal uptake was quantified by 2. The myocardial perfusion index was calculated as the sum of the scores of every segment divided by the total number of segments (normal value was 2). The myocardial perfusion score in this case was 30 and the myocardial perfusion index was 1.74.

## Discussion

In a patient with acute myocardial infarction efficiently revascularized in the therapeutic “window”, one month after the myocardial infarction, reperfusion was absent in the affected territory due to the impairment of microcirculation integrity, as evidenced by myocardial unopacified risk area. Therefore, myocardial recanalization was not followed by myocardial reperfusion. The information is useful in therapeutic management both on the short, medium, and long term [**2**]. The level of contrast homogeneity correlates with myocardial viability and recovery of myocardial function [**11**,**12**]. 

## Conclusions

Contrast echocardiography is a technique that improves endocardial tissue evaluation by ultrasound and can be used to enhance the Doppler evaluation and to assess the myocardial perfusion [**13**].

Left ventricular cavity ultrasound opacification by using microbubbles contributes to a more precise delineation of endocardial tissue and decreases variability in the detection of left ventricular wall motility abnormalities and ejection fraction analysis.

Microbubble contrast agents can be used to assess myocardial perfusion in stress and rest echocardiography. The prognostic value of myocardial perfusion study was proven by studies that have evaluated post-myocardial infarction recovery after revascularization maneuvers.
